# Recent Progress on Perovskite-Based Electrocatalysts for Efficient CO_2_ Reduction

**DOI:** 10.3390/molecules28248154

**Published:** 2023-12-18

**Authors:** Tong Wu, Lihua Zhang, Yinbo Zhan, Yilin Dong, Zheng Tan, Bowei Zhou, Fei Wei, Dongliang Zhang, Xia Long

**Affiliations:** Low Carbon College, Shanghai Jiaotong University, Shanghai 201306, China; wt1996@sjtu.edu.cn (T.W.); qlwlym@sjtu.edu.cn (L.Z.); zhanyinbo@sjtu.edu.cn (Y.Z.); 17805425648@sjtu.edu.cn (Y.D.); a.j.fikrytan@sjtu.edu.cn (Z.T.); bowei_sjtu@sjtu.edu.cn (B.Z.); weifei_sjtu@sjtu.edu.cn (F.W.); dongliang02@sjtu.edu.cn (D.Z.)

**Keywords:** perovskite, synthesis, CO_2_ electroreduction reaction, structural evolution, C_1_ and C_2+_ products

## Abstract

An efficient carbon dioxide reduction reaction (CO_2_RR), which reduces CO_2_ to low-carbon fuels and high-value chemicals, is a promising approach for realizing the goal of carbon neutrality, for which effective but low-cost catalysts are critically important. Recently, many inorganic perovskite-based materials with tunable chemical compositions have been applied in the electrochemical CO_2_RR, which exhibited advanced catalytic performance. Therefore, a timely review of this progress, which has not been reported to date, is imperative. Herein, the physicochemical characteristics, fabrication methods and applications of inorganic perovskites and their derivatives in electrochemical CO_2_RR are systematically reviewed, with emphasis on the structural evolution and product selectivity of these electrocatalysts. What is more, the current challenges and future directions of perovskite-based materials regarding efficient CO_2_RR are proposed, to shed light on the further development of this prospective research area.

## 1. Introduction

The booming development of industry and the economic level of modern society lead to an exponential growth in demand for energy, while the massive consumption of fossil fuels has resulted in some terrible environmental problems [1,2]. According to a report by the International Energy Agency (IEA), the total global greenhouse gas emissions in 2021 reached an equivalent of 40.8 billion tons of carbon dioxide (CO_2_), in which fuel combustion and industrial production accounted for ~89% [3,4]. Several strategies and policies such as the Paris Agreement [5,6] and China’s “3060 two carbon goals” have been proposed to tackle carbon emission issues, which have stimulated extensive fundamental studies on developing efficient materials and technologies to convert CO_2_ into value-added chemicals.

**Figure 1:**
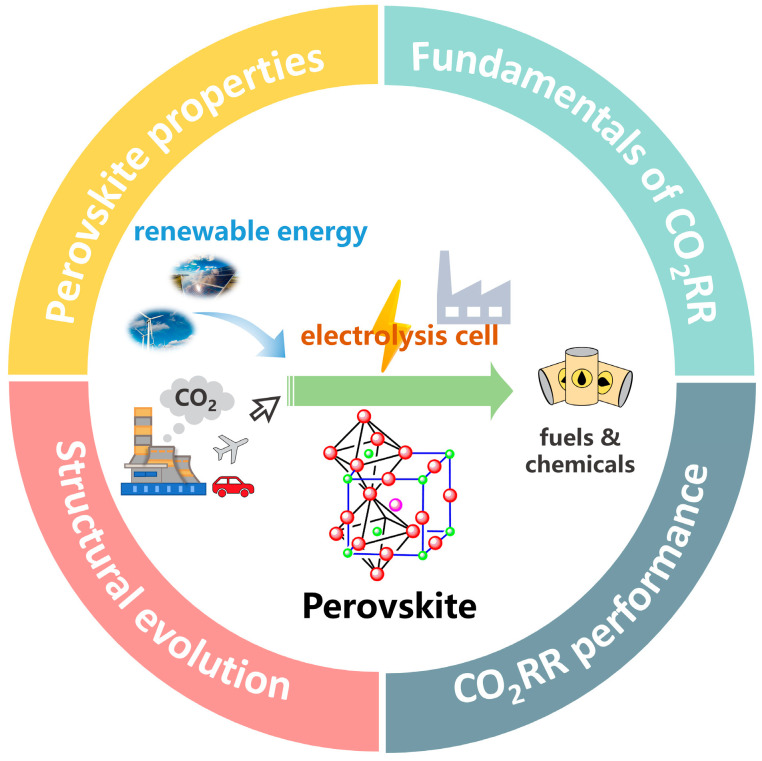
Schematic representation of the main content of this review.

The electrochemical reduction in CO_2_, activated by electricity from renewable energy sources such as wind, solar, etc., is one of the most promising methods for realizing the re-utilization of CO_2_ and dealing with the intermittency problem of renewable energy sources [7,8]. However, due to the stable chemical properties of CO_2_ molecules, the CO_2_ reduction process usually requires relatively harsh operation conditions, and the reaction pathways are greatly complicated, leading to varieties of products (e.g., CO, CH_4_, CH_3_OH, HCOOH, C_2_H_4_, C_2_H_6_, CH_3_CH_2_OH, CH_3_COOH, CH_3_CH_2_CH_2_OH, etc.) [9], despite the distinct equilibrium potentials of producing these chemicals (Table 1) [10]. Therefore, efficient catalysts with high activity, target product selectivity, and long-term stability are required to facilitate the CO_2_ reduction reaction (CO_2_RR) to meet the requirements of practical applications. Many significant achievements concerning noble-metal free electrocatalysts, especially inorganic perovskite oxides, which have some promising compositional and structural characters, have recently been obtained. Therefore, a timely review of this progress would surely attract a wide readership from both energy and material science search areas and contribute to the further advancement of CO_2_RR systems. Herein, as illustrated in Figure 1, we start with an introduction of the fundamental principles of CO_2_RR and the unique physicochemical properties of perovskites as electrocatalysts, followed by a detailed discussion on the applications of perovskite-based materials in the electrocatalysis of CO_2_RR, with emphasis on the investigation of structural evolution and product selectivity, using representative works published in the last few years. Finally, insights into the status, challenges and prospects for future research opportunities are provided. The search criteria used in this review included CO_2_RR/CO_2_ER and perovskite. Though most results were about solid oxide electrolyzers with high operation temperatures, our work mainly focuses on the low-temperature electrochemical studies of CO_2_RR catalyzed by perovskite-based materials. According to the search results, the works on utilizing perovskites as electrocatalysts for CO_2_RR are very limited, lacking a comprehensive review of the progress in this field. Herein, we provide such a review that systematically introduces the representative works published so far and discusses the key points deserving attention in future studies on electrochemical CO_2_RR processes catalyzed by perovskites-based materials, which would surely shed light on the design and modification of advanced perovskites-based electrocatalysts for efficient and sustainable CO_2_ conversion.

## 2. Fundamentals of CO_2_RR and Perovskite Oxides

### 2.1. Fundamentals of CO_2_RR

**Figure 2:**
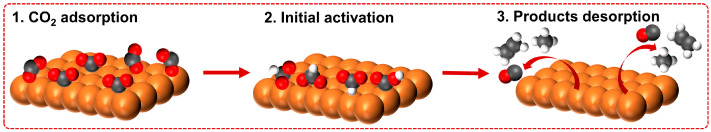
Three main steps on the catalyst surface of CO_2_RR processes. Black spheres represent carbon atoms; red spheres represent oxygen atoms; white spheres represent hydrogen atoms; orange atoms represent the CO_2_RR catalyst.

A CO_2_ reduction reaction (CO_2_RR) occurs at the interface of the tri-phase (CO_2_ gas, liquid electrolyte, and solid catalyst surface), involving multiple electron and proton transfer processes. Generally, three steps are required for CO_2_RR, as shown in Figure 2 [8,12,13]: (1) the CO_2_ molecules are adsorbed on the active sites on the catalyst surface; (2) the adsorbed CO_2_ molecules transform to a CO_2_^•−^ intermediate, and this initial electron transfer step is usually deemed the rate-determining step (RDS) of CO_2_RR; and (3) the generated products are desorbed and depart from the catalyst surface, making the active sites ready for the subsequent catalytic cycle. Nonetheless, the detailed reaction mechanisms are far more complicated than the abovementioned three basic steps, and the reaction pathways are distinct for different catalyst materials, even for the same product [14].

Laboratory-scale CO_2_RR is usually conducted in a three-electrode system, which is also the most widely used configuration in studies that investigate the catalytic performance of perovskite-based electrocatalysts. The system consists of a cathode (working electrode, where CO_2_RR occurs), an anode (usually platinum or nickel materials), and a reference electrode (Hg/HgO in an alkaline electrolyte, Ag/AgCl in a neutral or acidic electrolyte). Under the negative working potentials, CO_2_RR and the oxygen evolution reaction (OER) occur over the cathode and anode, respectively. Studies of CO_2_RR generally focus on the cathode, including both the catalytic materials and electrode structure. Herein, we only focus on the development of electrocatalysts for CO_2_RR. Generally, compared with metal catalysts, metal oxides have flexible and tunable structures, as well as interesting p–d coupling effects, which provide more abundant opportunities for tuning the electronic configuration of transition metal-based active sites and hence lead to promising catalytic properties. Among all the CO_2_RR catalysts that have been explored to date, perovskite-based materials exhibit some interesting characteristics and great potential in various electrochemical reactions [15,16,17,18,19,20].

### 2.2. Fundamentals of Perovskite Oxides

Perovskite is a family of compounds that have a general chemical formula of ABX_3_, the A-site elements could be rare-earth, alkaline-earth, or alkaline metals (e.g., Sr, La, Pr, Nd, Li, Na, Mg, Ca, etc.), while the B-site elements are transition metals (e.g., Ti, V, Mn, Fe, Co, Ni, Cu, etc.) that usually work as the active components during various catalytic reactions due to the existence of abundant d electrons and unfilled d orbitals. The anion X is usually oxygen for most of the perovskite-based CO_2_RR catalysts that will be discussed in this review article. Arising from the distinct atomic features of different A and B elements, the crystal structure of perovskites could be tuned by modulating the chemical compositions, which range from cubic to orthorhombic, tetragonal, or trigonal. In 1926, Goldschmidt proposed a relationship between the bond length of both A-X and B-X bonds to predict if a combination of a pair of cations could form the perovskite structure [21]. Then, this theory was optimized to use ionic radii as a substitute for bond length in order to expand the application range. The equation of modified Goldschmidt’s rule is displayed as follows:

$$t=\frac{r_{A}+r_{X}}{\sqrt{2}\left(r_{B}+r_{X}\right)}$$

where *t* is called the tolerance factor, *r_A_* is the radius of the A-site cation, *r_B_* is the radius of the B-site cation, and *r_X_* is the radius of the anion. The compound could be in an ideal perovskite structure when the tolerance factor is equal to precisely 1. Generally, the perovskite structure is stable when the tolerance factor is in the range of 0.75 to 1.00 [22]. If *t* lies in the approximately range of 0.9 to 1.0, the perovskite tends to exist in the ideal cubic structure. When *t* is lower than 0.9 but higher than 0.71, the octahedral structure will distort to break the cuboctahedral coordination and form a structure with a lower symmetry than the cubic one.

In addition to the simple ABX_3_ type, the family of perovskite materials also contains many other derivatives that show different atomic ratios, such as the Ruddlesden–Popper perovskite (RP-type) [22], Dion-Jacobson perovskite [23], Aurivillius perovskite [24], etc. All these perovskite derivatives can be realized by adjusting the thickness of perovskite units or inter-layer species. Regular and repeated phases can be synthesized when the structures are ordered, while disordered modules are non-stoichiometric. Such perovskites can be regarded as “layered materials”, which leads to their special physical structures and chemical characterizations. What is more, the special crystal structure of perovskites allows some elements to exist in unusual or mixed valence states with non-stoichiometric ratios of oxygen, leading to tunable and unique chemical properties that would further benefit the catalytic reactions. Actually, the positive roles of oxygen vacancies in perovskites for catalysis have been extensively investigated. It has been found that the moderate concentration of oxygen vacancies on the catalyst surface could effectively suppress the competitive hydrogen evolution reaction (HER) and also facilitate charge transfer during the CO_2_RR process [25].

The electrical conductivity of perovskites is another significant property when applied as an electrocatalyst. The RP-type perovskite materials were found to have high electrical conductivity due to the BO_6_ octahedra structure [26], which benefits the charge transfer process during electrochemical reactions [27]. Compared with the simple ABO_3_ perovskite, the RP-type perovskite (A_2_BO_4_) shows better electrical conductivity; moreover, the capability can be improved with the increasing n number because of the higher three-dimensional layered structure. In addition, the RP-type perovskite materials are stable enough under different pH conditions and oxidative potentials [28], making them promising electrocatalysts for many energy conversion reactions. In summary, the unique physicochemical properties of perovskite materials endow them with a broad application in various fields, such as energy conversion, storage, and electrochemical sensors [29,30].

### 2.3. Fabrication Methods of Perovskite Materials

In addition to chemical composition and crystal structure, particle size and microstructure also affect the performance of catalysts. Consequently, many approaches have been developed to synthesize perovskites with controlled particle size and morphology, such as wet chemistry methods, deposition-based approaches, templated-assisted synthesis, electrospinning, infiltration, exsolution, etc. In wet chemical synthesis, sol-gel processes are widely used to prepare nanosized perovskites [31,32]. In this method, citric acid [33] and ethylenediamine tetra-acetic acid [34] are often applied as the complexing agents to coordinate with the metal ions. A subsequent high-temperature calcination process should be carried out to remove the organics and facilitate the growth of perovskite crystals. Although the sol-gel method can produce purer-phase perovskites with higher surface areas when compared with the conventional solid-state method, the high-temperature calcination would inevitably lead to large agglomerated particles with a size of tens to hundreds of nanometers [35]. Template-assisted approaches have also been developed to synthesize perovskites with a porous microstructure [36]. Generally, a porous soft or hard template will be introduced in the preparation procedures, and then removed via calcination or chemical etching. The soft templates usually are self-assembled amphiphilic molecules [37,38] such as surfactants, and the hard ones can be silica-based molecular sieves [39,40] and organic polymethyl methacrylate (PMMA) [41,42]. The choice of templates should be based on the requirements for the pore size of the targeted structure of perovskites. Until now, perovskites with micropores, mesopores, three-dimensional ordered macropores, and hierarchical pores have been successfully prepared [43,44]. The porous microstructure could not only increase the surface area and hence provide more active sites but also provide convenient mass transfer channels, contributing to the greatly improved catalytic performance of perovskite-based materials for CO_2_RR.

## 3. Perovskite and Perovskite-Derived Catalysts for CO_2_RR

In this section, we summarize various perovskites or perovskite-derived materials, which were applied in the electrochemical CO_2_RR. CO_2_ can be converted into different valuable chemicals in both gas and liquid states. Generally, the products with one carbon (C_1_) include carbon monoxide, methane, methanol, and formate/formic acid, while ethylene, ethane, acetic acid, propanol, and other chemicals, with at least two carbon atoms, are classified into C_2+_ products. Due to the completely distinct reaction processes, e.g., the formation of C_2+_ requires C-C coupling, the design principles of catalysts for C_1_ and C_2+_ production via CO_2_RR are greatly different. Therefore, we classified the developed perovskite-based catalysts according to their main products in this section.

### 3.1. Perovskite-Based Catalysts for CO_2_RR Favoring C_1_ Products

As one of the most common products of CO_2_RR, CO is a widely used industrial resource for the synthesis of methanol and ammonia [45], as well as the feedstock of syngas with different H_2_-to-CO ratios. The relative studies have focused on enhancing faradaic efficiency (FE), which almost reached 100% with negligible H_2_ production [46,47,48]. Based on previous studies, it is well-known that a series of noble metals, including Au, Ag and Pd, have been proven to show remarkable CO_2_RR activity toward CO, with high FE and low overpotential [49,50,51]. For instance, the ultra-thin silver nanowires synthesized by Luo et al. [52] could realize the maximum FE (CO) of 99.3% with a quite small onset overpotential of 350 mV. In addition, some other active metals such as Zn [53,54], Co [55], and Ni [48] also attracted attention from researchers because the high cost of noble metals considerably restricts their industrial applications on a larger scale. Therefore, the specific element combinations in perovskites also help us to explore some efficient but inexpensive catalysts for CO_2_RR. In the study of Federico A’s team [56], a perovskite material with A-site substitution (La_0.5_Ba_0.5_CoO_3_) was prepared via the microwave irradiation method by using polycarbonate as the template. In this work, perovskites in a cubic structure, along with some other phases, were synthesized. The electrochemical and CO_2_RR tests were conducted in the H-cell and rotating ring-disc electrode (RRDE), respectively. All the products of CO_2_RR were C_1_ (CO and formate), and formate disappeared with the increase in reaction potential at −1.1 V vs. SHE. The density functional theory (DFT) calculations revealed that the CO_2_ molecules were adsorbed on the (001) and (110) planes of La_0.5_Ba_0.5_CoO_3_, where the surface oxygen sites were in a bend configuration. The generation of formate can be realized on both facets under the potential of −0.7 V and −0.8 V vs. SHE, while CO can only be generated on the (001) facet under more negative potentials.

Formate (in an alkaline environment)/formic acid (in a neutral or acidic environment) is another important C_1_ product of CO_2_RR and is an essential raw material in medical and chemical industries [57,58]. According to previous research, p-block elements including In, Sn, Pb, and Bi [59,60,61,62,63,64] and some copper-based catalysts [65] are highly reactive in converting CO_2_RR into formate/formic acid. The low cost and environmental friendliness of these active elements attract particular attention and a wide range of investigations. It is also noticeable that formate is produced in most studies instead of formic acid because most CO_2_RRs are carried out in alkaline electrolytes.

**Figure 3:**
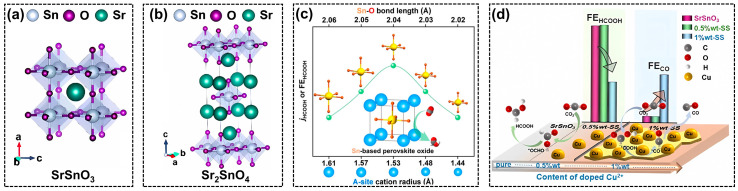
Crystal structures of (**a**) SrSnO_3_ and (**b**) Sr_2_SnO_4_, reprinted with permission from ref [66]. Copyright Royal Society of Chemistry, 2022; (**c**) structure–performance relationship of Ba_1−x_Sr_x_SnO_3_, reprinted with permission from ref. [72]. Copyright Wiley VCH GmbH, 2023; (**d**) doping effect of Cu^2+^ on SrSnO_3_ perovskite oxides, reprinted with permission from ref. [67]. Copyright Wiley VCH GmbH, 2022.

As a post-transition metal, tin (Sn) often acts as a B-site element in perovskite. In 2019, Huang et al. synthesized a one-dimensional SrSnO_3_ nanowires (NWs) via a facile hydrolysis method [66], which exhibited a much-enhanced selectivity toward formate production (FE~80%) with a long-term durability of 10 h, when compared with the traditional bulk perovskites and SnO_2_ nanoparticles. Based on a series of electrochemical characterizations, it was found that the stabilization of the intermediate CO_2_^•−^ promoted the kinetics for formate production, while the kinetics for HER can be inhibited over SrSnO_3_ NWs at the same time. However, the relationship between the NWs morphology and catalytic activity that is critically important remains unclear. The bulk SrSnO_3_ perovskite was also utilized as electrocatalysts for CO_2_RR in Zhang’s research [67]. The Cu^2+^ ions with different ratios were doped on the SrSnO_3_ (0.5 wt%-SS and 1 wt%-SS) to synthesize the final CO_2_RR catalysts as depicted in Figure 3d. Different from the studies over SS NWs that formate was the major product [66], the CO selectivity showed an obvious boost when the Cu^2+^ doping ratio reached to 1 wt%, with the highest FE (CO) of 49%. The product distribution indicated that the copper on the catalyst surface played the role of active species to generate CO, while the bulk SS could be active to generate formate, due to the different surface structure that changed the reaction pathway.

In the RP-type perovskite (A_2_BO_4_), the insertion of AO-interlayers into ABO_3_ can stabilize the crystal structure under negative potentials, avoiding the catalyst deactivation due to the surface amorphization and structural reconstruction [68,69,70]. Gong’s group prepared an RP-type Sr_2_SnO_4_ perovskite through a solid-state approach and managed to solve the instability problem of SrSnO_3_ during CO_2_RR [71]. Though the active elements in perovskites were found to be reduced into a metallic state under the negative working potential of CO_2_RR, they found that Sr_2_SnO_4_ could stably work at –1.08 V vs. RHE for more than 24 h, due to the much-improved structural durability of RP perovskite (Figure 3b) when compared with the normal SrSnO_3_ (Figure 3a). The insertion of the SrO layer led to the strong interaction between perovskite layers and inhibited the structural reconstruction during CO_2_RR.

It is also reported that tuning A-site cations in perovskite influences the coordination environment of B-site elements, modifying the physicochemical properties of the catalysts. Therefore, another Sn-based Ba_1–x_Sr_x_SnO_3_ perovskite with different A-site element ratios was synthesized via a high-energy ball-milling process [72]. With the change of Ba:Sr ratios, the average cation radius decreased from 1.61 to 1.44 Å, leading to the shortening of the Sn-O bonds from 2.06 to 2.02 Å (Figure 3c). The precise regulation of Sn-O bonds strengthened the bond covalency and shifted the total band center closer to the Fermi level. The much-enhanced catalytic performance suggests that A-site modulation is indeed an efficient approach to idealizing the electronic structure, electron transfer property, adsorption of reaction intermediates, and other important features of perovskite-based catalysts.

**Figure 4:**
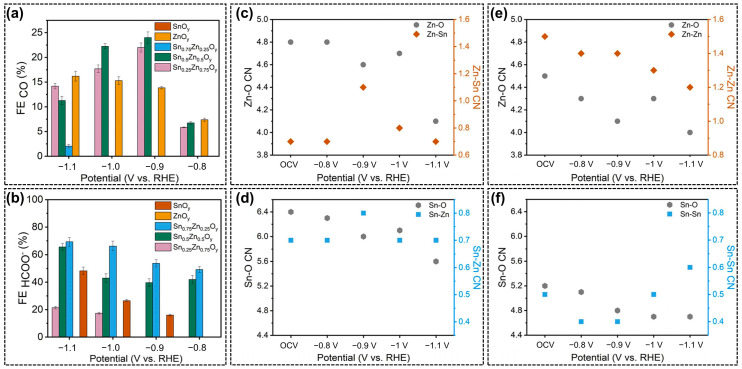
(**a**) FE (CO) and (**b**) FE (formate) over Sn_x_Zn_y_O_z_ catalysts; Different coordination numbers (CN) calculated based on in-situ extended X-ray absorption fine structure (EXAFS) results for (**c**,**d**) Sn_0.5_Zn_0.5_O_y_, (**e**) ZnO_y_, and (**f**) SnO_y_, measured under electrochemical CO_2_RR at potentials of open circuit voltage, 0.8, 0.9, 1.0, and 1.1 V. vs. RHE, reprinted with permission from ref. [73]. Copyright Elsevier Ltd., 2022.

The exsolution of B-site elements in perovskite oxides under a reductive environment (negative potential or reductive atmosphere) is an inevitable phenomenon, and such a structural evolution is pivotal for the identification of active sites and reaction mechanisms. Bedford et al. [73] applied a novel biomineralization approach for the synthesis of perovskite-type tin-zinc oxides (TZO), which exhibited a porous microstructure with abundant defects and amorphous phases, rather than normal perovskite oxides with specific crystal structures. The as-prepared Sn_x_Zn_y_O_z_ exhibited good catalytic activity toward CO_2_RR with the major products of formate and CO. What is more, as exhibited in Figure 4a,b, the product selectivity was found to vary with Sn:Zn ratios due to the distinct capability of Sn and Zn in the formation of CO. The coordination numbers during CO_2_RR demonstrated that the electronic configurations and local structures of metal components depended on the applied cathodic potentials over different Sn_x_Zn_y_O_z_ catalysts (Figure 4c–f), generating highly active sites of oxygen vacancies that determined the CO_2_RR performance. The DFT modeling results also proved that oxygen vacancy can facilitate the interaction between intermediates and undercoordinated Sn and Zn sites to tune the selectivity toward formate.

**Figure 5:**
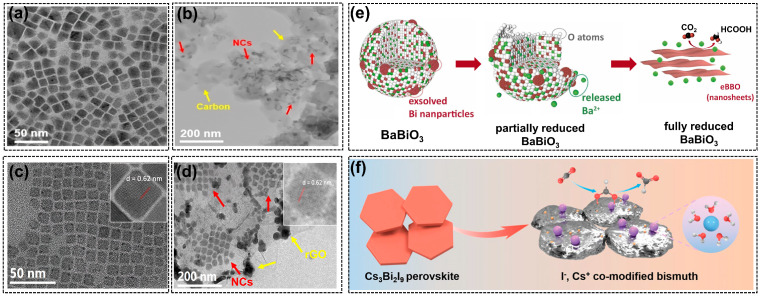
The transmission electron microscope (TEM) images of (**a**) Cs_3_Bi_2_Br_9_ NCs and (**b**) Cs_3_Bi_2_Br_9_/Carbon black composite, reprinted with permission from ref. [78]. Copyright Wiley VCH GmbH, 2022; TEM images of (**c**) CsPbI_3_ NCs and (**d**) CsPbI_3_/rGO composite, reprinted with permission from ref [79]. Copyright Springer Ltd., 2023, red arrows and yellow arrows in (**b**,**d**) point to perovskite nanocubes and carbon materials, respectively; (**e**) the structural evolution of BaBiO_3_ perovskite during CO_2_RR, reprinted with permission from ref. [76]. Copyright Elsevier Ltd., 2022; (**f**) the conversion of Cs_3_Bi_2_I_9_ nanocrystals to bismuth with I^−^ and Cs^+^ dual modification, reprinted with permission from ref [75]. Copyright Wiley VCH GmbH, 2023.

In addition to Sn, bismuth (Bi) is another active species for converting CO_2_ into C_1_ products. Previous research proved that Bi species are more favorable to generating formate in aqueous solutions [74]. The advantages of Bi include environmental friendliness, low cost, high biocompatibility, and being inert to hydrogen evolution [75]. In the study of Luo’s team [76], the electricity-driven reconstructions of a BaBiO_3_ (BBO) perovskite were emphasized. The experimental results showed that an irreversible structural and phase transformation can be observed during CO_2_RR, as displayed in Figure 5e. The B-site Bi in pristine BBO exsolved from the perovskite crystals under cathodic potentials. Then, the re-nucleation and growth of dissolved Bi particles led to the complete transformation of BBO from the monoclinic phase to Bi nanosheets with atomic-scale thickness, acting as the real active species during CO_2_RR. Notably, the authors compared the structural reconstruction of the BBO catalyst with another typical RP-type perovskite (La_2_CuO_4_) that was also active in the CO_2_RR [77]. La_2_O_3_, the reduction product of La_2_CuO_4_, is insoluble in aqueous electrolytes; however, due to the solubility of BaO, the cleavage of Ba-O and Bi-O bonds resulted in almost complete A-site element loss. The ionic form, Ba^2+^, can also enhance the adsorption of carbonate intermediates, thereby promoting formate production. This study revealed the real structure and active species of perovskites during CO_2_RR again, and the electrochemical reduction process can be regarded as a catalyst synthesis method using perovskite material as the precursor.

Consequently, a perovskite precursor with halide and alkali metal compositions was prepared by a hot-injection method and showed a hexagonal Cs_3_Bi_2_I_9_ nanocrystal [75]. The pre-catalyst was successfully converted into Bi crystals with halide and alkali metal dual modification (Figure 5f). The trace amounts of I^–^ and Cs^+^ ions were found to be located on the Bi surface and outer Helmholtz layer, respectively, which affected the catalytic processes. There are also many works on the combination of halide perovskites with carbon materials to improve their stability. For example, Cs_3_Bi_2_Br_9_ was mixed with carbon black (Figure 5a,b) to form a composite efficient for CO_2_RR in an acidic electrolyte (HBr) [78]. The in-situ tests and characterizations of electrodes after CO_2_RR proved that the Cs_3_Bi_2_Br_9_/C composites exhibited remarkable structural durability due to the quick electron transport and fast CO_2_ reduction rate, which leads to a smaller degradation effect. This method also works well for stabilizing perovskite materials with lead (Pb) and indium (In) in their B-sites. For example, Wang et al. reported a metal halide perovskite (CsPbI_3_) that combined with reduced graphene oxide (rGO), which effectively tackled the poor phase stability of inorganic halide perovskite [79]. Generally, with the addition of rGO, the perovskite nanocrystals would be wrapped and stabilized by rGO (Figure 5c,d), avoiding the formation of inactive Pb defects or Pb nanoparticles usually observed after the CO_2_RR in pristine CsPbI_3_. What is more, the electron density near the perovskite/rGO interface was also regulated, contributing to the enhanced durability and selectivity of formate formation.

Additionally, it is noteworthy that halide perovskites demonstrate a broader range of applications in the field of CO_2_ conversion compared to perovskite oxides, particularly in terms of CO_2_RR via photoelectrochemical (PEC) processes. PEC reduction technology can transform CO_2_ directly into a variety of chemicals within integrated photo absorber-electrocatalyst systems, driven by both solar and electrical energy [80,81], thereby mitigating excessive reliance on external power sources. Among the semiconductor materials for PEC CO_2_ reduction, lead-based halide perovskites have garnered significant attention because of their appealing photoelectronic properties, including a defect-tolerant band structure, high photoluminescence efficiency, a wide absorption range, and a tunable band gap expanding the whole visible range [82,83,84]. Due to toxicity and long-term instability, lead-based halide perovskites are usually combined with other materials during PEC CO_2_ reduction. Huang et al. [85] modified the CsPbBr_3_ (CPB) perovskite with fluorine doping, Nafion solution, and Au coating. Such an interface-engineering approach can greatly facilitate light adsorption and charge transfer when compared with the pristine photocathode. In another study by Luo et al. [86], PbS nanoparticles were uniformly anchored on CPB nanocrystals with the assistance of an amino acid as a capping ligand. The introduction of PbS significantly boosted the charge transfer, leading to enhanced CO and CH_4_ production rates of 2.94 and 0.36 μmol cm^−2^ h^−1^, respectively. Besides this, a lead-free halide perovskite of CsAgBr_2_ was also successfully synthesized and exhibited superior CO_2_ reduction performance, with around 14 μmol g^−1^ h^−1^ of CO yield and 50% FE [87]. These studies underscored the practicability of directly using inorganic halide perovskites in PEC CO_2_ reduction.

Though there are still many uncertainties regarding the structure–performance relationship of perovskite derivatives for CO_2_ reduction, especially the underlying reasons for the stability, it could be concluded that perovskites could be good precursors for preparing the metallic catalysts that are active for CO_2_RR. The rational coupling of perovskites with other materials such as carbon and organic capping ligands could efficiently enhance their stability during the reaction process.

The CO_2_RR with methane as the main product is most likely to be realized on copper-based materials, and copper is one of most utilized B-site elements for forming the perovskite structure. In the study of Xia’ et al. [77], the RP-type La_2_CuO_4_ perovskite was applied in CO_2_RR to produce methane. The structural evolution during CO_2_RR was emphasized to play the key role in the methanation process. The surface layers of La_2_CuO_4_ were partially reduced under negative potentials to form metallic Cu^0^ and La_2_O_3_, generating the heterostructure of Cu/La_2_CuO_4_. Such a hetero-interface can optimize the surface adsorption of CO_2_ and charge transfer, leading to considerable methanation activity, with an FE (methane) of 56.3% at −1.4 V vs. RHE. A-site elements in perovskites are usually supposed to be inactive during catalytic reactions, and the candidates include lanthanide elements (La, Ce, Pr, etc.) and rare earth metals (Mg, Ca, Ba, etc.). A recent report [88] chose the alkaline-earth element of Ca as an A-site element to synthesize the perovskite oxides (Ca_2_CuO_3_). The calcium at the A-site caused Ca_2_CuO_3_ to feature a strong basic strength and a remarkable capability for CO_2_ chemisorption, compared with the non-basic Sr_2_CuO_3_ and La_2_CuO_4_. The leaching problem of Ca^2+^ ions also occurred over Ca_2_CuO_3_ perovskite. However, the leaching can help to form uncoordinated copper sites, which were beneficial for the hydrogenation of *CO and *CHO intermediates to generate *CH_2_O, leading to great methanation activity with extremely high current densities and relatively low overpotentials.

In addition to Cu-based materials, some other perovskite catalysts also exhibited good capability of producing methane. It was reported for the first time that lead halide perovskite (CsPbBr_3_) nanocrystals showed high activity in CO_2_RR for producing methane and CO [89]. The water-dispersible CsPbBr_3_ exhibited an ultrahigh catalytic stability of 350 h in H-cells, where the FE (CO) and FE (methane) were 40% and 32%, respectively. A diverse range of perovskite materials (LaCoO_3_, LaCrO_3_ LaMnO_3_, LaFeO_3_, etc.) were also prepared and examined for electrochemical methanation [90], and the LaCoO_3_ perovskite exhibited the highest methanation activity among all the samples. The O 2p-band center was proposed as an activity descriptor that can rationalize activity and selectivity for CO_2_RR, which influences the CO_ads_ binding energy of the catalyst to enhance CH_4_ selectivity. The studies about electrochemical methanation using perovskite materials as catalysts are much fewer than the papers focused on producing CO and formate, and the catalytic performance is far below satisfactory. Consequently, there are still plenty of strategies and there is much development room for perovskite-based catalysts to improve methane selectivity by rationally designing both the chemical composition and microstructure of the perovskite-based catalytic materials.

### 3.2. Perovskite-Based Catalysts for C_2+_ Production

Common C_2+_ products of CO_2_RR include ethylene, ethanol, acetic acid, propanol, etc., which generally possess higher energy densities and economic value than C_1_ products [91]. Similar to methane production, copper-based perovskite materials are also widely used to facilitate *CO dimerization and hydrogenation, which are regarded as the key pathways for generating C_2+_ products [92].

**Figure 6:**
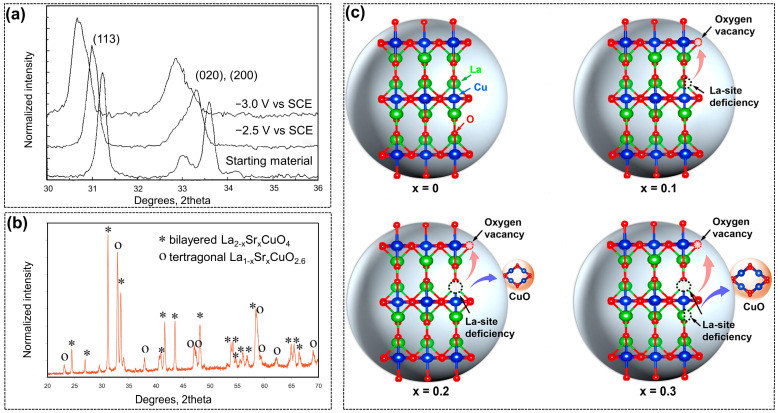
(**a**) XRD patterns of La_1.8_Sr_0.2_O_4_ before and after CO_2_RR, reprinted with permission from ref. [93]. Copyright The Electrochemical Society Inc, 1993; (**b**) La_2-x_Sr_x_CuO_4_ synthesized by Mignard et al., reprinted with permission from ref. [95]. Copyright Elsevier Ltd., 2014; (**c**) illustration of crystal structure properties of L_2_C, L_1.9_C, L_1.8_C, and L_1.7_C, reprinted with permission from ref. [96]. Copyright Wiley VCH GmbH, 2021.

In 1993, Schwartz [93] applied perovskites as electrocatalysts in CO_2_RR for the first time, and the main products were found to be various alcohols. The RP-type perovskite with different A and B elements was tested over gas diffusion electrodes. The results indicated all the non-copper-containing perovskite materials were inactive toward CO_2_RR, while the total FE for alcohols including methanol, ethanol, and n-propanol could reach around 40% over La_1.8_Sr_0.2_O_4_ at 180 mA/cm^−2^. Furthermore, the valence changes of copper ions in the perovskites were confirmed via X-ray diffraction (XRD) patterns before and after CO_2_RR tests, as shown in Figure 6a, demonstrating the structural evolution of perovskite crystals, and the active sites could be the mixture of Cu^0^ and Cu^n+^. This ground-breaking work proved the potential activity of copper-based perovskite in CO_2_RR, and more importantly, it reminded subsequent researchers that the structural evolution during the reaction should be emphasized.

Decades after the first paper, the RP-type copper-based perovskite was investigated in CO_2_RR again by various research teams [94,95,96]. Mignard et al. [95] further investigated RP-type La_2-x_Sr_x_CuO_4_ for CO_2_RR with modified testing conditions, including a much enlarged applied potential range, reaction temperatures from 2 to 40 °C, and CO_2_ pressures from 1 to 43 bar. The main products were unexpected to be the hydrocarbons of methane and ethylene, rather than alcohols, as in Schwartz’s work. The differences were further analyzed from several aspects such as electrolyte recirculation, impurities, and perovskite compositions. The different perovskite crystals could be the determinant. The synthesized La_1.8_Sr_0.2_O_4_ exhibited two crystal phases of (LaSr)CuO_4_ and (LaSr)CuO_2.6_ (Figure 6b), even though prepared with the same procedure. The ratios between these two perovskite phases could be vital to the product distribution of CO_2_RR; however, this was without the support of experimental data. Despite the distinctions in results, this study still displayed the essential influences of reaction conditions and devices on the CO_2_RR process. Further, the structural change of La_2_CuO_4_ under negative potentials was systematically investigated based on a series of in-situ and ex-situ experiments [94]. According to the electrochemical cyclic voltammetry curves, it was found that the RP-type La_2_CuO_4_ underwent a two-step electroreduction, which was attributed to Cu^2+^ to Cu_2_O and Cu_2_O to metallic Cu^0^, respectively. The in-situ formed Cu^+^ in the perovskite was considered as the active site for CO_2_RR to produce ethylene. In addition, the influence of A-site cation in La_2_CuO_4_ was also fully investigated [96]. Nonstoichiometric La_2–x_CuO_4–δ_ (x = 0, 0.1, 0.2, and 0.3) with different La amounts was synthesized to introduce vacancies into the perovskite structure. As depicted in Figure 6c, when the La deficiency concentration was relatively low (x < 0.1), a certain quantity of oxygen vacancies could efficiently facilitate the C_2+_ formation and suppress the competing HER. Nonetheless, too much of an La deficiency (x > 0.1) would result in the phase separation of the perovskite and form CuO/perovskite hybrids, and the CuO was favorable for methane and H_2_ production. In short, by simply controlling the cation defects, researchers could realize the tunable catalytic selectivity of defected La_2–x_CuO_4–δ_ hybrids toward CO_2_RR to generate C_2+_ products.

**Figure 7:**
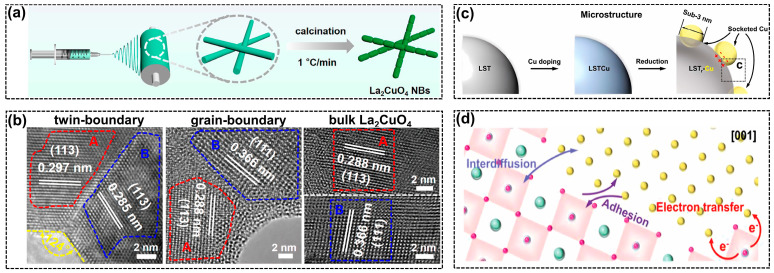
(**a**) Illustration of electrospinning approach to prepare La_2_CuO_4_ NBs, reprinted with permission from ref. [69]. Copyright ACS Publications, 2021; (**b**) the high-resolution transmission electron microscope (HRTEM) images of grain boundaries in La_2_CuO_4_ with different micro-morphologies, reprinted with permission from ref. [69]. Copyright ACS Publications, 2021; (**c**) schematic illustrations of microstructure evolutions during the LSTr-Cu formation, reprinted with permission from ref. [100]. Copyright Wiley VCH GmbH, 2022; (**d**) the SMSIs for the LSTr-Cu catalyst, reprinted with permission from ref. [100]. Copyright Wiley VCH GmbH, 2022.

Grain boundaries and interface engineering are emerging techniques to optimize the adsorption of reaction intermediates on the catalysts, thereby enhancing the selectivity for particular products [97,98]. Such strategies also work well over perovskites to facilitate C_2+_ hydrocarbon production via CO_2_RR. The electrospinning approach (Figure 7a) was applied to synthesize La_2_CuO_4_ catalysts with different microscopic morphologies [69]. With the increase in heating rate during calcination, the perovskite morphology changed from nano bamboos (NBs) to nanorods (NRs), and finally to bulk perovskite. The optimized perovskite NBs exhibited symmetric twin boundaries of the (113) facet, as depicted in Figure 7b, which induced a strain effect that facilitated charge transfer and C-C coupling, leading to the high FE (ethylene) of 60% at −1.0 V vs. RHE. However, a high FE (CO) of 91% was observed over the bulk perovskite without regular grain boundaries. What is more, the introduction of twin boundaries seemed to be helpful for stabilizing the perovskite structure based on the characterization results of spent catalysts. The grain boundaries can also be generated by an in-situ electroreduction treatment [99]. The La_2_CuO_4_-derived copper catalyst presented multiple GBs composed of different nanocrystallites, including Cu (111) and Cu_2_O (111). With the assistance of CO_2_, the La oxides can form soluble La(CO_3_)_3_ and further lead to the complete phase evolution from a single-crystal perovskite to diverse grains of copper species, such as metallic Cu^0^ and copper oxides. The high-density grain boundaries generated more defective sites, which were beneficial for the strong binding of CO_ads_, therefore promoting C-C coupling to realize a high C_2+_ selectivity of 80.3%.

Similar to electroreduction, thermal reduction is another strategy to construct active copper species derived from perovskites (Figure 7c). The strong metal-support interactions (SMSIs) were introduced by in-situ generated copper nanoparticles that uniformly dispersed over La_0.4_Sr_0.4_Ti_0.9_O_3−δ_ (LST) support, forming the LSTr-Cu catalyst with sub-3 nm copper nanoparticles on the surface [100]. Figure 7d illustrates that the SMSIs effect not only modulated the electronic properties but facilitated the adsorption of key reaction intermediates. More importantly, strong adhesion between the surface Cu atoms and LST backbones inhibited the migration of active copper clusters, enhancing the resistance to active site degradation. Although the stability test was conducted in H-cells with a low current density of 15 mA/cm^2^, the positive influence of SMSIs could definitely be demonstrated according to the stable FE (CO_2_RR). Moreover, the perovskite was found to be reactive in producing other C_2+_ products such as oxalate. The production of oxalate via CO_2_RR usually suffers from high energy barriers and slow kinetics [74]. Surprisingly, a recently published paper found that the lead-based perovskite material (PbSnO_3_/C) exhibited excellent activity with a low overpotential and high FE (oxalate) of 85.1% [101]. Though the underlying reasons are still obscure, this provides promising opportunities for modifying the physicochemical properties of perovskite materials to realize controlled CO_2_RR and target products such as oxalate.

## 4. Summary and Outlook

Perovskite materials with the general formula of ABX_3_ have abundant catalytically active transition-metal ions in B-sites, which have found extensive applications in diverse electrochemical reactions and energy conversion devices over an extended period. However, the utilization of perovskite-based catalysts for efficient CO_2_RR remains at the initial stage, and it needs a timely and comprehensive review to delve into the underlying problems and challenges according to the reported studies and hence promote the further development of this research area. In this review, we encapsulate the recent progress of perovskites and their derivatives as electrocatalysts for CO_2_RR, starting with the introduction of fundamentals of CO_2_RR and perovskite materials. Then, the pivotal and representative studies of perovskites as CO_2_RR catalysts are comprehensively discussed according to the major products of CO_2_RR (Table 2).

By analyzing and discussing these representative works, we found that the catalytic activity, selectivity and stability of perovskites and their derivatives for CO_2_RR are strongly related to their chemical composition, crystal structure, micro-morphology, etc. In general, the active B-site metals determine the product selectivity. For instance, Sn-, Pb-, Bi-, and In-based perovskites mainly generate C_1_ products such as formate and CO, while Cu-based perovskites could be good candidates for the formation of methane or C_2+_ products. As for the influence of crystal structure, an RP-type perovskite was found to possess better structural stability because the insertion of AO-interlayers could stabilize the ABO_3_ structure under negative working potentials of CO_2_RR. In addition, the crystal structure could also be tuned via A-site substitution. The mismatch between the ion radius of different A-site elements would result in the formation of lattice distortion and vacancies and even generate a second phase that contributes to the change of product selectivity.

What is more, most of the perovskite materials usually underwent structural evolution during CO_2_RR processes. Some preparation methods such as electrospinning were developed to fabricate perovskites with special micro-morphologies such as nanowires, nanorods, and nano bamboos, which demonstrated much enhanced structural stability. Further, the structural evolution phenomenon was utilized to synthesize high-active metallic catalysts by using a perovskite as a precursor. Both of these directions can realize the controlled preparation of perovskite-based materials with suitable microstructures. In addition, the real active sites could also evolve during the structural reconstruction. Therefore, the precise determination of active sites and their evolution pathways should be exploited in order to reveal the structure–performance relationship of the perovskite-based electrocatalysts toward CO_2_RR.

Last but not least, the advancements and possible research directions to address the existing limitations are summarized and proposed below:

1. The pivotal factor in assessing a catalyst material lies in its catalytic activity. As for the production of C_1_ products, the recently developed noble metal-based or even transition metal-based catalysts can reach an FE (CO) close to 100% [102,103,104,105], and other lead- and tin-based catalysts can also reach an FE (formate) higher than 90% [106,107]. However, the selectivity of perovskite-based catalysts toward CO_2_RR is far below these levels, particularly in C_2+_ production. Thus, there is large space for further improving the activity and selectivity of perovskite-based catalysts for producing high-value C_2+_ products, which would be realized by precisely modulating the physicochemical characteristics of active sites.
2. The active transition-metal ions in perovskites would be reduced under the negative potential condition during CO_2_RR. Though a few papers improved the stability of their perovskite-based materials during CO_2_RR, the reduction processes could be challenging to observe under the small current densities applied in their studies. Consequently, there is a pressing need for further investigations on the possible structure evolution of perovskites under high current densities.
3. Moreover, in-situ technologies are essential to provide real-time information on the evolution of active sites during the CO_2_RR process. For instance, in-situ XRD and X-ray photoelectron spectroscopy (XPS) could provide direct experimental results to observe the change in crystal structure and metal valences; in-situ Raman and Fourier transform infrared (FTIR) are also crucial for revealing the catalytic mechanism [108,109]. Additionally, theoretical simulation of the reaction mechanisms of CO_2_RR over perovskites also needs to take the structural change into consideration, because the selection of active facets during CO_2_RR profoundly impacts the accuracy of the calculations.
4. Until now, most studies have focused on applying perovskite oxides for CO_2_RR, while metal oxides have to face a series of issues such as instability under negative potentials and acidic electrolytes. Therefore, inorganic perovskites incorporate other anions such as halide elements (F^−^, Br^−^, I^−^, etc.) [110,111], which have been reported to possess the capability of regulating the electronic properties of copper and enhancing its selectivity toward C_2+_ products, so they deserve further exploration.

## Figures and Tables

**Table 1:** Electrochemical reactions in CO_2_RR under equilibrium potentials [11].

Electrolyte Conditions	Reactions	*E*^0^/(V vs. RHE)	Products
/	2H ^+^ + 2e^−^ → H_2_	0	hydrogen evolution reaction (HER)
/	xCO_2_ + nH ^+^ + ne^−^ → product + yH_2_O	/	CO_2_RR
Alkaline (pH: 8~14)	CO_2_ + 2H ^+^ + 2e^−^ → H ^+^ + HCOO^–^	−0.12	formate
	CO_2_ + 2H ^+^ + 2e^−^ → CO (g) + H_2_O	−0.10	carbon monoxide
	CO_2_ + 6H ^+^ + 6e → CH_3_OH (aq) + H_2_O	0.03	methanol
	CO_2_ + 4H ^+^ + 4e^−^ → C (s) + 2H_2_O	0.21	carbon
	CO_2_ + 8H ^+^ + 8e^−^ → CH_4_ (g) + 2H_2_O	0.17	methane
	2CO_2_ + 8H ^+^ + 8e^−^ → H ^+^ + CH_3_COO^–^ + 2H_2_O	0.11	acetate
	2CO_2_ + 10H ^+^ + 10e^−^ → CH_3_CHO (aq) + 3H_2_O	0.06	acetaldehyde
	2CO_2_ + 12H ^+^ + 12e^−^ → C_2_H_5_OH (aq) + 3H_2_O	0.09	ethanol
	2CO_2_ + 2H ^+^ + 12e^−^ → C_2_H_4_ (g) + 4H_2_O	0.08	ethylene
	2CO_2_ + 14H ^+^ + 14e^−^ → C_2_H_6_ (g) + 4H_2_O	0.14	ethane
	3CO_2_ + 18H ^+^ + 18e^−^ → C_3_H_7_OH (aq) + 5H_2_O	0.10	propanol
Acidic (pH: 0~7)	CO_2_ + 2H ^+^ + 2e^−^ → HCOOH (aq)	−0.12	formic acid
	2CO_2_ + 8H ^+^ + 8e^−^ → CH_3_COOH (aq) + 2H_2_O	0.11	acetic acid

**Table 2:** Summary of the representative studies discussed in this review.

Perovskite Catalyst	Major Products	FE of Main Products	Reference
Cs_3_Bi_2_Br_9_/carbon black	formic acid	92%	[78]
SrSnO_3_ nanowires	formate	~80%	[66]
Sr_2_SnO_4_	formate	83.7%	[71]
Sn_a_Zn_b_O_y_	formate	~70%	[73]
BaBiO_3_	formate	98.7%	[76]
Ba_1−x_Sr_x_SnO_3_	formate	90.9%	[96]
CsPbI_3_/rGO	formate	>92%	[79]
Cs3Bi_2_I_9_	formate	98.2%	[75]
La_2_CuO_4_	methane	56.3%	[77]
Ca_2_CuO_3_	methane	51.7%	[88]
La_1.8_Sr_0.2_CuO_4_	methane and ethylene	Not mentioned	[95]
Cu-SrSnO_3_	CO and formate	89% (formate) over 0.5 wt% Cu-SrSnO_3_; 49% (CO) over 1 wt% Cu-SrSnO_3_	[64]
CsPbBr_3_	methane and CO	32% for methane, 40% for CO	[89]
La_0.5_Ba_0.5_CoO_3_	CO	85%	[56]
A_1.8_A_0.2_CuO_4_ (A = La, Pr, and Gd; A’ = Sr and Th)	alcohols	~40%	[93]
La_2_CuO_4_	ethylene	40.3%	[94]
La_2_CuO_4_ nanobamboos	ethylene	60%	[69]
reduced La_0.4_Sr_0.4_Ti_0.9_O_3−δ_-Cu	C_2+_ products	54.9%	[100]
La_2–x_CuO_4-δ_	C_2+_ products	41.5%	[96]
La_2_CuO_4_ derived-Cu	C_2+_ products	80.3%	[99]
PbSnO_3_/C	oxalate	85.1%	[101]

## Data Availability

No new data were created.

## References

[1] Shen M., Kong F., Tong L., Luo Y., Yin S., Liu C., Zhang P., Wang L., Chu P.K., Ding Y. (2022). Carbon capture and storage (CCS): Development path based on carbon neutrality and economic policy. Carbon Neutrality.

[2] Zheng Y., Ma M., Shao H. (2023). Recent advances in efficient and scalable solar hydrogen production through water splitting. Carbon Neutrality.

[3] Agency I.E. (2023). CO_2_ Emissions in 2022.

[4] Agency I.E. (2021). Global Energy Review: CO_2_ Emissions in 2021.

[5] Bai X.F., Chen W., Wang B.Y., Feng G.H., Wei W., Jiao Z., Sun Y.H. (2017). Recent progress on electrochemical reduction of carbon dioxide. Acta Phys.-Chim. Sin..

[6] Van Vuuren D.P., Stehfest E., Gernaat D.E.H.J., van den Berg M., Bijl D.L., de Boer H.S., Daioglou V., Doelman J.C., Edelenbosch O.Y., Harmsen M. (2018). Alternative pathways to the 1.5  °C target reduce the need for negative emission technologies. Nat. Clim. Chang..

[7] Handoko A.D., Wei F., Jenndy, Yeo B.S., Seh Z.W. (2018). Understanding heterogeneous electrocatalytic carbon dioxide reduction through operando techniques. Nat. Catal..

[8] Birdja Y.Y., Pérez-Gallent E., Figueiredo M.C., Göttle A.J., Calle-Vallejo F., Koper M.T.M. (2019). Advances and challenges in understanding the electrocatalytic conversion of carbon dioxide to fuels. Nat. Energy.

[9] Yan Y., Ke L., Ding Y., Zhang Y., Rui K., Lin H., Zhu J. (2021). Recent advances in Cu-based catalysts for electroreduction of carbon dioxide. Mater. Chem. Front..

[10] Nitopi S., Bertheussen E., Scott S.B., Liu X.Y., Engstfeld A.K., Horch S., Seger B., Stephens I.E.L., Chan K., Hahn C. (2019). Progress and perspectives of electrochemical CO reduction on copper in aqueous electrolyte. Chem. Rev..

[11] Woldu A.R., Huang Z., Zhao P., Hu L., Astruc D. (2022). Electrochemical CO_2_ reduction (CO_2_RR) to multi-carbon products over copper-based catalysts. Coord. Chem. Rev..

[12] Huang J., Buonsanti R. (2019). Colloidal nanocrystals as heterogeneous catalysts for electrochemical CO_2_ conversion. Chem. Mater..

[13] Zhu P., Wang H. (2021). High-purity and high-concentration liquid fuels through CO_2_ electroreduction. Nat. Catal..

[14] Yan T., Chen X., Kumari L., Lin J., Li M., Fan Q., Chi H., Meyer T.J., Zhang S., Ma X. (2023). Multiscale CO_2_ electrocatalysis to C_2+_ products: Reaction mechanisms, catalyst design, and device fabrication. Chem. Rev..

[15] Xiao J., Liu L., Zhang D., De Marco N., Lee J.W., Lin O., Chen Q., Yang Y. (2017). The emergence of the mixed perovskites and their applications as solar cells. Adv. Energy Mater..

[16] Retuerto M., Calle-Vallejo F., Pascual L., Lumbeeck G., Fernandez-Diaz M.T., Croft M., Gopalakrishnan J., Peña M.A., Hadermann J., Greenblatt M. (2019). La_1.5_Sr_0.5_NiMn_0.5_Ru_0.5_O_6_ double perovskite with enhanced ORR/OER bifunctional catalytic activity. ACS Appl. Mater. Interfaces.

[17] Zhu Y., Zhou W., Zhong Y., Bu Y., Chen X., Zhong Q., Liu M., Shao Z. (2017). A perovskite nanorod as bifunctional electrocatalyst for overall water splitting. Adv. Energy Mater..

[18] Sun Y., Li R., Chen X., Wu J., Xie Y., Wang X., Ma K., Wang L., Zhang Z., Liao Q. (2021). A-site management prompts the dynamic reconstructed active phase of perovskite oxide OER catalysts. Adv. Energy Mater..

[19] Xu X., Chen Y., Zhou W., Zhu Z., Su C., Liu M., Shao Z. (2016). A perovskite electrocatalyst for efficient hydrogen evolution reaction. Adv. Mater..

[20] Li D., Zhang D., Lim K.-S., Hu Y., Rong Y., Mei A., Park N.-G., Han H. (2021). A review on scaling up perovskite solar cells. Adv. Funct. Mater..

[21] Tilley R.J. (2016). Perovskites: Structure-Property Relationships.

[22] Xu X.M., Pan Y.L., Zhong Y.J., Ran R., Shao Z.P. (2020). Ruddlesden-Popper perovskites in electrocatalysis. Mater. Horiz..

[23] Yukta, Parikh N., Chavan R.D., Yadav P., Nazeeruddin M.K., Satapathi S. (2022). Highly efficient and stable 2D Dion Jacobson/3D perovskite heterojunction solar cells. ACS Appl. Mater. Interfaces.

[24] Kendall K.R., Navas C., Thomas J.K., zur Loye H.-C. (1996). Recent developments in oxide Ion conductors: Aurivillius phases. Chem. Mater..

[25] Li L., Zhao Z., Hu C., Yang P., Yuan X., Wang Y., Zhang L., Moskaleva L., Gong J. (2020). Tuning oxygen vacancies of oxides to promote electrocatalytic reduction of carbon dioxide. ACS Energy Lett..

[26] Lee D., Lee H.N. (2017). Controlling oxygen mobility in Ruddlesden-Popper oxides. Materials.

[27] Toda K., Kameo Y., Kurita S., Sato M. (1996). Crystal structure determination and ionic conductivity of layered perovskite compounds NaLnTiO_4_ (Ln = rare earth). J. Alloys Compd..

[28] May K.J., Carlton C.E., Stoerzinger K.A., Risch M., Suntivich J., Lee Y.-L., Grimaud A., Shao-Horn Y. (2012). Influence of oxygen evolution during water oxidation on the surface of perovskite oxide catalysts. J. Phys. Chem. Lett..

[29] Xu X., Su C., Shao Z. (2021). Fundamental understanding and application of Ba_0.5_Sr_0.5_Co_0.8_Fe_0.2_O_3−δ_ perovskite in energy storage and conversion: Past, present, and future. Energy Fuels.

[30] He J., Xu X., Li M., Zhou S., Zhou W. (2023). Recent advances in perovskite oxides for non-enzymatic electrochemical sensors: A review. Anal. Chim. Acta.

[31] Peng X., Feng S., Lai S., Liu Z., Gao J., Javanbakht M., Gao B. (2022). Structural engineering of rare-earth-based perovskite electrocatalysts for advanced oxygen evolution reaction. Int. J. Hydrogen Energy.

[32] Xu X., Wang W., Zhou W., Shao Z. (2018). Recent advances in novel nanostructuring methods of perovskite electrocatalysts for energy-related applications. Small Methods.

[33] Han X., Hu Y., Yang J., Cheng F., Chen J. (2014). Porous perovskite CaMnO_3_ as an electrocatalyst for rechargeable Li-O_2_ batteries. Chem. Commun..

[34] Jung J.I., Jeong H.Y., Lee J.S., Kim M.G., Cho J. (2014). A bifunctional perovskite catalyst for oxygen reduction and evolution. Angew. Chem..

[35] Chen C.-F., King G., Dickerson R.M., Papin P.A., Gupta S., Kellogg W.R., Wu G. (2015). Oxygen-deficient BaTiO_3−x_ perovskite as an efficient bifunctional oxygen electrocatalyst. Nano Energy.

[36] Cui X., Wu T., Gai D., Yang C., Ding Y., Zhao P. (2023). Enhancement of perovskites performance for coal tar decomposition by pore structure and acid-base modification. Fuel.

[37] Lu F., Wang Y., Jin C., Li F., Yang R., Chen F. (2015). Microporous La_0.8_Sr_0.2_MnO_3_ perovskite nanorods as efficient electrocatalysts for lithium-air battery. J. Power Sources.

[38] Lee Y.C., Peng P.Y., Chang W.S., Huang C.M. (2014). Hierarchical meso-macroporous LaMnO_3_ electrode material for rechargeable zinc–air batteries. J. Taiwan Inst. Chem. Eng..

[39] Wang Y., Cui X., Li Y., Chen L., Shu Z., Chen H., Shi J. (2013). High surface area mesoporous LaFe_x_Co_1−x_O_3_ oxides: Synthesis and electrocatalytic property for oxygen reduction. Dalton Trans..

[40] Yang Y., Zhou W., Liu R., Li M., Rufford T.E., Zhu Z. (2015). In Situ tetraethoxysilane-templated porous Ba_0. 5_Sr_0. 5_Co_0. 8_Fe_0. 2_O_3−δ_ perovskite for the oxygen evolution reaction. ChemElectroChem.

[41] Sun Y., Zhang Y., Yang Y., Chen J., Hua B., Shi Y., Wang C., Luo J. (2017). Smart tuning of 3D ordered electrocatalysts for enhanced oxygen reduction reaction. Appl. Catal. B Environ..

[42] Oh M.Y., Lee J.J., Zahoor A., Gnana kumar G., Nahm K.S. (2016). Enhanced electrocatalytic activity of three-dimensionally-ordered macroporous La_0.6_Sr_0.4_CoO_3−δ_ perovskite oxide for Li-O_2_ battery application. RSC Adv..

[43] Qiu P., Ma B., Hung C.-T., Li W., Zhao D. (2019). Spherical mesoporous materials from single to multilevel architectures. Acc. Chem. Res..

[44] Su X., Sun Y., Jin L., Zhang L., Yang Y., Kerns P., Liu B., Li S., He J. (2020). Hierarchically porous Cu/Zn bimetallic catalysts for highly selective CO_2_ electroreduction to liquid C_2_ products. Appl. Catal. B Environ..

[45] Ham Y.S., Choe S., Kim M.J., Lim T., Kim S.-K., Kim J.J. (2017). Electrodeposited Ag catalysts for the electrochemical reduction of CO_2_ to CO. Appl. Catal. B Environ..

[46] Hao Y., Hu F., Zhu S., Sun Y., Wang H., Wang L., Wang Y., Xue J., Liao Y.-F., Shao M. (2023). MXene-regulated metal-oxide interfaces with modified intermediate configurations realizing nearly 100% CO_2_ electrocatalytic conversion. Angew. Chem. Int. Ed..

[47] Liu S., Sun C., Xiao J., Luo J.-L. (2020). Unraveling structure sensitivity in CO_2_ electroreduction to near-unity CO on silver nanocubes. ACS Catal..

[48] Kim Y.E., Ko Y.N., An B.-S., Hong J., Jeon Y.E., Kim H.J., Lee S., Lee J., Lee W. (2023). Atomically dispersed nickel coordinated with nitrogen on carbon nanotubes to boost electrochemical CO_2_ reduction. ACS Energy Lett..

[49] Sun D., Xu X., Qin Y., Jiang S.P., Shao Z. (2020). Rational design of Ag-based catalysts for the electrochemical CO_2_ reduction to CO: A review. ChemSusChem.

[50] Kauffman D.R., Alfonso D., Matranga C., Qian H., Jin R. (2012). Experimental and computational investigation of Au25 clusters and CO_2_: A unique interaction and enhanced electrocatalytic activity. J. Am. Chem. Soc..

[51] Zhu W., Kattel S., Jiao F., Chen J.G. (2019). Shape-controlled CO_2_ electrochemical reduction on nanosized Pd hydride cubes and octahedra. Adv. Energy Mater..

[52] Liu S., Wang X.-Z., Tao H., Li T., Liu Q., Xu Z., Fu X.-Z., Luo J.-L. (2018). Ultrathin 5-fold twinned sub-25 nm silver nanowires enable highly selective electroreduction of CO_2_ to CO. Nano Energy.

[53] Luo W., Zhang J., Li M., Züttel A. (2019). Boosting CO production in electrocatalytic CO_2_ reduction on highly porous Zn catalysts. ACS Catal..

[54] Li C., Shen G., Zhang R., Wu D., Zou C., Ling T., Liu H., Dong C., Du X. (2019). Zn nanosheets coated with a ZnS subnanometer layer for effective and durable CO_2_ reduction. J. Mater. Chem. A.

[55] Aljabour A., Coskun H., Apaydin D.H., Ozel F., Hassel A.W., Stadler P., Sariciftci N.S., Kus M. (2018). Nanofibrous cobalt oxide for electrocatalysis of CO_2_ reduction to carbon monoxide and formate in an acetonitrile-water electrolyte solution. Appl. Catal. B Environ..

[56] Cardona J.F.Z., Sacanell J., Barral M.A.A., Vildosola V., Viva F. (2022). CO_2_ reduction on a nanostructured La_0.5_Ba_0.5_CoO_3_ perovskite: Electrochemical characterization and DFT calculations. J. CO2 Util..

[57] An L., Chen R. (2016). Direct formate fuel cells: A review. J. Power Sources.

[58] Calabrese M., Russo D., di Benedetto A., Marotta R., Andreozzi R. (2023). Formate/bicarbonate interconversion for safe hydrogen storage: A review. Renew. Sustain. Energy Rev..

[59] Yang F., Elnabawy A.O., Schimmenti R., Song P., Wang J., Peng Z., Yao S., Deng R., Song S., Lin Y. (2020). Bismuthene for highly efficient carbon dioxide electroreduction reaction. Nat. Commun..

[60] Li J., Li J., Liu X., Chen J., Tian P., Dai S., Zhu M., Han Y. (2021). Probing the role of surface hydroxyls for Bi, Sn and In catalysts during CO_2_ reduction. Appl. Catal. B Environ..

[61] Deng W., Zhang L., Li L., Chen S., Hu C., Zhao Z.J., Wang T., Gong J. (2019). Crucial role of surface hydroxyls on the activity and stability in electrochemical CO_2_ reduction. J. Am. Chem. Soc..

[62] Pander J.E., Lum J.W.J., Yeo B.S. (2019). The importance of morphology on the activity of lead cathodes for the reduction of carbon dioxide to formate. J. Mater. Chem. A.

[63] Lu X., Wu Y., Yuan X., Wang H. (2019). An integrated CO_2_ electrolyzer and formate fuel cell enabled by a reversibly restructuring Pb-Pd bimetallic catalyst. Angew. Chem. Int. Ed..

[64] Zhang Z., Liu C., Brosnahan J.T., Zhou H., Xu W., Zhang S. (2019). Revealing structural evolution of PbS nanocrystal catalysts in electrochemical CO_2_ reduction using in situ synchrotron radiation X-ray diffraction. J. Mater. Chem. A.

[65] Li J., Meng C., Gu J., Wang H., Dai R., Sha H., Zhu H. (2022). High faradaic efficiency of CO_2_ conversion to formic acid catalyzed by Cu_2_O hollow-dices. Carbon Neutrality.

[66] Pi Y., Guo J., Shao Q., Huang X. (2019). All-inorganic SrSnO_3_ perovskite nanowires for efficient CO_2_ electroreduction. Nano Energy.

[67] Wang Y.Y., Wang Z.L., Wang D., Mao J.J., Zhang C.C., Zhang Y. (2022). Revealing the doping effect of Cu^2+^ on SrSnO_3_ perovskite oxides for CO_2_ electroreduction. ChemElectroChem.

[68] Chen Y., Li H., Wang J., Du Y., Xi S., Sun Y., Sherburne M., Ager J.W., Fisher A.C., Xu Z.J. (2019). Exceptionally active iridium evolved from a pseudo-cubic perovskite for oxygen evolution in acid. Nat. Commun..

[69] Wang J., Cheng C., Huang B., Cao J., Li L., Shao Q., Zhang L., Huang X. (2021). Grain-boundary-engineered La_2_CuO_4_ perovskite nanobamboos for efficient CO_2_ reduction reaction. Nano Lett..

[70] Zhu C., Tian H., Huang B., Cai G., Yuan C., Zhang Y., Li Y., Li G., Xu H., Li M. (2021). Boosting oxygen evolution reaction by enhanced intrinsic activity in Ruddlesden-Popper iridate oxides. Chem. Eng. J..

[71] Zhao J., Zhang P., Li L., Yuan T., Gao H., Zhang G., Wang T., Zhao Z.-J., Gong J. (2022). SrO-layer insertion in Ruddlesden–Popper Sn-based perovskite enables efficient CO_2_ electroreduction towards formate. Chem. Sci..

[72] Chen M., Chang K., Zhang Y., Zhang Z., Dong Y., Qiu X., Jiang H., Zhu Y., Zhu J. (2023). Cation-radius-controlled Sn−O bond length boosting CO_2_ electroreduction over Sn-based perovskite oxides. Angew. Chem. Int. Ed..

[73] Jiang J., Huang B., Daiyan R., Subhash B., Tsounis C., Ma Z., Han C., Zhao Y., Effendi L.H., Gallington L.C. (2022). Defective Sn-Zn perovskites through bio-directed routes for modulating CO_2_RR. Nano Energy.

[74] Wang G., Chen J., Ding Y., Cai P., Yi L., Li Y., Tu C., Hou Y., Wen Z., Dai L. (2021). Electrocatalysis for CO_2_ conversion: From fundamentals to value-added products. Chem. Soc. Rev..

[75] Luo Y., Chen S., Zhang J., Ding X., Pan B., Wang L., Lu J., Cao M., Li Y. (2023). Perovskite-derived bismuth with I^−^ and Cs^+^ dual modification for high-efficiency CO_2_-to-formate electrosynthesis and Al-CO_2_ batteries. Adv. Mater..

[76] Zhu M., Zhang B., Gao M.-R., Sui P.-F., Xu C., Gong L., Zeng H., Shankar K., Bergens S., Luo J. (2022). Electrochemically reconstructed perovskite with cooperative catalytic sites for CO_2_-to-formate conversion. Appl. Catal. B Environ..

[77] Chen S., Su Y., Deng P., Qi R., Zhu J., Chen J., Wang Z., Zhou L., Guo X., Xia B.Y. (2020). Highly selective carbon dioxide electroreduction on structure-evolved copper perovskite oxide toward methane production. ACS Catal..

[78] Wang Y., Wang C., Wei Y., Wei F., Kong L., Feng J., Lu J., Zhou X., Yang F. (2022). Efficient and selective electroreduction of CO_2_ to HCOOH over bismuth-based bromide perovskites in acidic electrolytes. Chem.—A Eur. J..

[79] Hoang M.T., Han C., Ma Z., Mao X., Yang Y., Madani S.S., Shaw P., Yang Y., Peng L., Toe C.Y. (2023). Efficient CO_2_ reduction to formate on CsPbI_3_ nanocrystals wrapped with reduced graphene oxide. Nano-Micro Lett..

[80] Chen J., Yin J., Zheng X., Ait Ahsaine H., Zhou Y., Dong C., Mohammed O.F., Takanabe K., Bakr O.M. (2019). Compositionally screened eutectic catalytic coatings on halide perovskite photocathodes for photoassisted selective CO_2_ reduction. ACS Energy Lett..

[81] Zhang N., Long R., Gao C., Xiong Y. (2018). Recent progress on advanced design for photoelectrochemical reduction of CO_2_ to fuels. Sci. China Mater..

[82] Pan A., Ma X., Huang S., Wu Y., Jia M., Shi Y., Liu Y., Wangyang P., He L., Liu Y. (2019). CsPbBr_3_ perovskite nanocrystal grown on MXene nanosheets for enhanced photoelectric detection and photocatalytic CO_2_ reduction. J. Phys. Chem. Lett..

[83] Zhang X., Wu X., Liu X., Chen G., Wang Y., Bao J., Xu X., Liu X., Zhang Q., Yu K. (2020). Heterostructural CsPbX_3_-PbS (X = Cl, Br, I) quantum dots with tunable Vis-NIR dual emission. J. Am. Chem. Soc..

[84] Luo B., Li F., Xu K., Guo Y., Liu Y., Xia Z., Zhang J.Z. (2019). B-site doped lead halide perovskites: Synthesis, band engineering, photophysics, and light emission applications. J. Mater. Chem. C.

[85] Zhang X., Tang R., Sun H., Yang W., Liang W., Li F., Zheng R., Huang J. (2023). Synergistically interface-engineered inorganic halide perovskite photocathodes for photoelectrochemical CO_2_ reduction. Energy Fuels.

[86] Wu X., Xu R., Li X., Zeng R., Luo B. (2022). Amino acid-assisted preparation of homogeneous PbS/CsPbBr_3_ nanocomposites for enhanced photoelectrocatalytic CO_2_ reduction. J. Phys. Chem. C.

[87] Makani N.H., Singh M., Paul T., Sahoo A., Nama J., Sharma S., Banerjee R. (2022). Photoelectrocatalytic CO_2_ reduction using stable lead-free bimetallic CsAgBr_2_ halide perovskite nanocrystals. J. Electroanal. Chem..

[88] Xu Z., Peng C., Luo G., Yang S., Yu P., Yan S., Shakouri M., Wang Z., Sham T.-K., Zheng G. (2023). High-rate CO_2_-to-CH_4_ electrosynthesis by undercoordinated Cu sites in alkaline-earth-metal perovskites with strong basicity. Adv. Energy Mater..

[89] Chen K., Qi K., Zhou T., Yang T., Zhang Y., Guo Z., Lim C.-K., Zhang J., Žutic I., Zhang H. (2021). Water-dispersible CsPbBr_3_ perovskite nanocrystals with ultra-stability and its application in electrochemical CO_2_ reduction. Nano-Micro Lett..

[90] Hwang J., Akkiraju K., Corchado-García J., Shao-Horn Y. (2019). A perovskite electronic structure descriptor for electrochemical CO_2_ reduction and the competing H_2_ evolution reaction. J. Phys. Chem. C.

[91] Chang B., Pang H., Raziq F., Wang S., Huang K.-W., Ye J., Zhang H. (2023). Electrochemical reduction of carbon dioxide to multicarbon (C_2+_) products: Challenges and perspectives. Energy Environ. Sci..

[92] Fan L., Liu C.-Y., Zhu P., Xia C., Zhang X., Wu Z.-Y., Lu Y., Senftle T.P., Wang H. (2022). Proton sponge promotion of electrochemical CO_2_ reduction to multi-carbon products. Joule.

[93] Schwartz M., Cook R.L., Kehoe V.M., MacDuff R.C., Patel J., Sammells A.F. (1993). Carbon dioxide reduction to alcohols using perovskite-type electrocatalysts. J. Electrochem. Soc..

[94] Singh R.P., Arora P., Nellaiappan S., Shivakumara C., Irusta S., Paliwal M., Sharma S. (2019). Electrochemical insights into layered La_2_CuO_4_ perovskite: Active ionic copper for selective CO_2_ electroreduction at low overpotential. Electrochim. Acta.

[95] Mignard D., Barik R.C., Bharadwaj A.S., Pritchard C.L., Ragnoli M., Cecconi F., Miller H., Yellowlees L.J. (2014). Revisiting strontium-doped lanthanum cuprate perovskite for the electrochemical reduction of CO_2_. J. CO2 Util..

[96] Zhu J., Wang Y., Zhi A., Chen Z., Shi L., Zhang Z., Zhang Y., Zhu Y., Qiu X., Tian X. (2022). Cation-deficiency-dependent CO_2_ electroreduction over copper-based Ruddlesden-Popper perovskite oxides. Angew. Chem. Int. Ed..

[97] Pang Y., Li J., Wang Z., Tan C.S., Hsieh P.-L., Zhuang T., Liang Z., Zou C., Wang X., De Luna P. (2019). Efficient electrocatalytic conversion of carbon monoxide to propanol using fragmented copper. Nat. Catal..

[98] Dinh C., Burdyny T., Kibria M.G., Seifitokaldani A., Gabardo C.M., García de Arquer F.P., Kiani A., Edwards J.P., De Luna P., Bushuyev O.S. (2018). CO_2_ electroreduction to ethylene via hydroxide-mediated copper catalysis at an abrupt interface. Science.

[99] Niu Z., Chi L., Wu Z., Yang P., Fan M., Gao M. (2023). CO_2_-assisted formation of grain boundaries for efficient CO-CO coupling on a derived Cu catalyst. Natl. Sci. Open.

[100] Li Y., Liu F., Chen Z., Shi L., Zhang Z., Gong Y., Zhang Y., Tian X., Zhang Y., Qiu X. (2022). Perovskite-socketed sub-3 nm copper for enhanced CO_2_ electroreduction to C_2+_. Adv. Mater..

[101] Cheng Y., Hou P., Pan H., Shi H., Kang P. (2020). Selective electrocatalytic reduction of carbon dioxide to oxalate by lead tin oxides with low overpotential. Appl. Catal. B Environ..

[102] Chung M.W., Cha I.Y., Ha M.G., Na Y., Hwang J., Ham H.C., Kim H.-J., Henkensmeier D., Yoo S.J., Kim J.Y. (2018). Enhanced CO_2_ reduction activity of polyethylene glycol-modified Au nanoparticles prepared via liquid medium sputtering. Appl. Catal. B Environ..

[103] Hall A.S., Yoon Y., Wuttig A., Surendranath Y. (2015). Mesostructure-induced selectivity in CO_2_ reduction catalysis. J. Am. Chem. Soc..

[104] Jeong H.-Y., Balamurugan M., Choutipalli V.S.K., Jeong E.-S., Subramanian V., Sim U., Nam K.T. (2019). Achieving highly efficient CO_2_ to CO electroreduction exceeding 300 mA cm^−2^ with single-atom nickel electrocatalysts. J. Mater. Chem. A.

[105] Wen C.F., Mao F., Liu Y., Zhang X.Y., Fu H.Q., Zheng L.R., Liu P.F., Yang H.G. (2020). Nitrogen-stabilized low-valent Ni motifs for efficient CO_2_ electrocatalysis. ACS Catal..

[106] Li D., Wu J., Liu T., Liu J., Yan Z., Zhen L., Feng Y. (2019). Tuning the pore structure of porous tin foam electrodes for enhanced electrochemical reduction of carbon dioxide to formate. Chem. Eng. J..

[107] Fan M., Garbarino S., Botton G.A., Tavares A.C., Guay D. (2017). Selective electroreduction of CO_2_ to formate on 3D [100] Pb dendrites with nanometer-sized needle-like tips. J. Mater. Chem. A.

[108] Gong Y., He T. (2023). Gaining deep understanding of electrochemical CO_2_RR with in situ/operando techniques. Small Methods.

[109] Zou Y., Wang S. (2021). An investigation of active sites for electrochemical CO_2_ reduction reactions: From in situ characterization to rational design. Adv. Sci..

[110] Cai R., Sun M., Ren J., Ju M., Long X., Huang B., Yang S. (2021). Unexpected high selectivity for acetate formation from CO_2_ reduction with copper based 2D hybrid catalysts at ultralow potentials. Chem. Sci..

[111] Cai R., Sun M., Yang F., Ju M., Chen Y., Gu M.D., Huang B., Yang S. (2023). Engineering Cu(I)/Cu(0) interfaces for efficient ethanol production from CO_2_ electroreduction. Chem.

